# Investigating the relationships between physical activity, mindful self-compassion, and well-being among Chinese adolescents: the Exercise Self-Esteem Model Revised with Self-Compassion 2.0

**DOI:** 10.1080/00049530.2026.2620254

**Published:** 2026-02-01

**Authors:** Ming Yu Claudia Wong, Ana Cláudia Mesquita-Garcia, Hong Wang Fung

**Affiliations:** a Department of Health and Physical Education, The Education University of Hong Kong, Hong Kong, China; b Interdisciplinary Center for Studies in Palliative Care, School of Nursing, Federal University of Alfenas, Alfenas, Brazil; c School of Nursing, The Hong Kong Polytechnic University, Hong Kong, China

**Keywords:** Physical activity, self-compassion, mindfulness, EXSEM-SC model, well-being

## Abstract

**Background:**

Physical activity is widely recognised as beneficial for well‑being, yet the psychological mechanisms underlying this relationship, particularly those involving mindfulness and self‑compassion, remain insufficiently examined. The Exercise and Self‑Esteem Model with Self‑Compassion (EXSEM‑SC) highlights the roles of exercise self‑efficacy and body compassion in fostering self‑compassion, but it does not address the contributions of mindfulness and interoceptive body awareness in mitigating self‑criticism associated with discomfort, inadequacy, or doubt during activity.

**Purpose:**

To address this gap, the present cross‑sectional study conducted in Hong Kong integrated the Mindful and Compassionate Awareness Scale for Physical Activity (MCA‑PA) to investigate associations among physical activity, MCA‑PA, self‑compassion, and well‑being.

**Methods and Results:**

Data were collected from 414 secondary school students (44.9% female; *M* = 14.95 years, SD = 1.60), and structural equation modelling confirmed that MCA‑PA and self‑compassion mediated the relationship between physical activity and well‑being, with no gender differences (CFI = 0.97, SRMR = 0.039, RMSEA = 0.065, 90% CI [0.00, 0.13]).

**Conclusion:**

These findings underscore the importance of mindfulness and self‑compassion in explaining how physical activity enhances well‑being, suggesting that health promotion programs in educational and broader contexts should incorporate practices that cultivate mindful and compassionate awareness to strengthen psychological outcomes and support healthy behaviours.

## KEY POINTS

**What is already known about this topic:**

1. Physical activity is consistently associated with improved subjective well-being, quality of life, and mental health across various populations.
2. Self-compassion is positively associated with physical activity behaviours, motivation, and self-efficacy, supporting engagement and maintenance of physical activity
3. The Exercise Self-Esteem Model Revised with Self-Compassion (EXSEM-SC) demonstrates that exercise self-efficacy and body compassion mediate the relationship between physical activity and self-compassion, but it overlooks mindfulness and interoceptive awareness.

**What this topic adds:**

1. The Mindful and Compassionate Awareness Scale for Physical Activity (MCA-PA) effectively mediates the relationship between physical activity and well-being, emphasising the role of mindful and compassionate engagement during exercise.
2. Moderate- and low-intensity physical activities, such as walking or yoga, foster greater mindful and compassionate awareness compared to high-intensity activities, promoting self-compassion and psychological well-being.
3. Health promotion programs should integrate mindfulness and self-compassion training with physical activity interventions to enhance psychological well-being, particularly in adolescent populations.

## Background

### Physical activity and well-being

Physical activity is widely recognised for its health benefits, and systematic reviews and meta-analyses have examined how physical activity influences subjective well-being, quality of life, and mental health across different age groups and populations. Physical activity is consistently associated with improved subjective well-being and quality of life in healthy individuals, including older adults and school-age children (Andermo et al., 2020). Physical activity is also more strongly linked to positive affect, resilience, and psychological well-being than to reductions in negative affect or symptoms of depression and anxiety (Neill et al., 2020). While the success of physical activity interventions seems not to be heavily influenced by the type, duration, or increase in physical activity, certain moderators (such as frequency and session duration) might affect specific mental health outcomes (Andermo et al., 2020). However, a large-scale cross-sectional survey involving 67,770 adults in Wales, UK, identified a curvilinear association between moderate-to-vigorous physical activity and health-related quality of life (Alzahrani, 2022). In contrast, research also supports the fact that a reverse curvilinear dose-response relationship exists between physical exercise and depression, characterised by steeper gradients at lower activity levels. This pattern suggests that the transition from inactivity to activity yields the most significant benefits. The research indicates that if less active adults had met current physical activity guidelines, 11.5% of depression cases could have been averted. This highlights the public health significance of promoting even modest improvements in physical activity (Pearce et al., 2022).

### Self-compassion and physical activity

Self‑compassion, defined as treating oneself with kindness during setbacks (Neff, 2003), has also been linked to motivation, described as “a sensitivity to suffering in self and others with a commitment to try to alleviate and prevent it” (Gilbert, 2014). This motivational orientation is context‑dependent; as Gilbert (2019) notes, compassionate behaviours vary according to function, such that “a firefighter smelling smoke, a mother empathically soothing her child after a bad dream, and a consoling nurse will feel and behave very differently”. Hence, self‑compassion has gained attention as a psychological resource that may support engagement in, and maintenance of, physical activity. Research has examined the relationships among self‑compassion, physical activity behaviours, motivation, mental health, and intervention outcomes (Sirois et al., 2015; Wong et al., 2021). A meta‑analysis reported positive associations between self‑compassion (including intervention effects) and both physical health (*r* = .18) and health behaviour (*r* = .26; Phillips & Hine, 2021). Among children and adolescents, self‑compassion is associated with physical‑activity‑related self‑regulation, motivation, affect, and self‑efficacy (Kullman et al., 2024). These findings suggest that a healthy view of the self is positively linked to identified, integrated, and intrinsic motivation for exercise, as well as task‑oriented goal setting (Magnus et al., 2010). Conversely, the influence of physical activity on self‑compassion has been less extensively explored. Meta‑analyses have summarised a significant association between physical activity interventions and increases in self‑compassion (M. Y. C. Wong et al., 2021), although most interventions included were light‑intensity activities (e.g., yoga, tai chi). The EXSEM‑SC model has demonstrated a direct relationship between physical activity and self‑compassion, as well as mediating effects of self‑efficacy and body compassion (M. Y. C. Wong et al., 2023, 2021). However, existing evidence has not yet clarified how different intensities of physical activity relate to self‑compassion.

It is essential to acknowledge the role of physical activity in self-compassion, as highlighted by existing research. However, the emphasis on the relationship between dosage and the type of physical activity appears insufficient to substantiate the role of physical activity in fostering self-compassion. Self-compassion involves a complex combination of perceptions related to self-concept, self-response, self-evaluation, and locus of control. It is therefore essential to investigate the internal processes that occur during physical activity. For example, the State Mindfulness Scale for Physical Activity (SMS‑PA; Ullrich-French et al., 2017) is a validated instrument for assessing mindfulness during physical activity, with a focus on present‑moment awareness of mental and bodily sensations. In contrast, the Mindful and Compassionate Awareness Scale for Physical Activity (MCA‑PA; Wong et al., under review) is a 20‑item instrument that extends the SMS‑PA‑2 by integrating self‑compassion and body‑compassion constructs. Specifically, the MCA‑PA was developed by adding keywords and items related to self‑compassion to the original SMS‑PA‑2 framework, resulting in a four‑factor structure (CFI = 0.99, TLI = 0.98) that captures not only mindful awareness but also compassionate responses to physical and mental experiences during physical activity. This integration reflects the natural progression from mindful awareness to compassionate engagement, whereby practitioners develop self‑soothing strategies and constructive ways of managing negative thoughts beyond non‑judgemental awareness (Neff & Dahm, 2015). By combining compassionate awareness with mindfulness, a compassionate mindset enhances the ability to recognise personal flaws and hardships within the context of shared humanity, thereby mitigating self‑blame and over‑identification during physical activity (Neff & Dahm, 2015).

### Theoretical frameworks and the research gap

The theoretical foundation for examining the relationship between physical activity, self‑compassion, and well‑being has been significantly advanced by the EXSEM‑SC model (M. Y. C. Wong et al., 2021, 2023), which illustrates the conceptual links between physical activity and self‑compassion by incorporating exercise self‑efficacy and body compassion. The EXSEM‑SC model retains the variable of exercise self‑efficacy while substituting physical competence and acceptance with body compassion, emphasising the role of compassionate awareness and acceptance of the body in fostering psychological well‑being. However, although the EXSEM‑SC highlights the pathways through which exercise self‑efficacy and body compassion contribute to self‑compassion and, ultimately, well‑being, a notable limitation remains: the model does not adequately account for the dynamic influence of mindfulness and interoceptive body awareness in this relationship. Research indicates that mindfulness meditation can significantly modify perceptions of one’s emotions and bodily signals (Neff & Dahm, 2015). Furthermore, body responsiveness which includes both body awareness and disconnection from the body, appears to mediate the relationship between mindfulness practices and outcomes such as life satisfaction, suggesting that interoceptive awareness may be a fundamental mechanism through which mindfulness exerts its beneficial effects on psychological well‑being (Sünbül & Özcan, 2022).

Specifically, while body compassion encompasses acceptance and kindness towards one’s body, it primarily focuses on attitudinal aspects rather than the active, moment-to-moment awareness of bodily sensations and movements cultivated through mindfulness. Similarly, exercise self-efficacy, as conceptualised within the EXSEM-SC, predominantly emphasizes the belief in one’s ability to perform physical activities, often neglecting the experiential and reflective dimensions of the activity itself. This narrow focus on performance outcomes overlooks the importance of mindfully sensing the body during physical activity and the capacity to accept imperfections or challenges that arise during exercise. For instance, issues like physical discomfort, perceived deficiencies, or instances of self-doubt that arise during physical exertion are frequently confronted with self-criticism or judgement (Roychowdhury, 2021), which can obstruct the cultivation of self-compassion.

The MCA-PA addresses these limitations by capturing the integrated, state-level experience of mindfulness and compassion occurring simultaneously during physical activity. This integration is theoretically and practically important: mindfulness provides the foundation of present-moment, non-judgemental awareness of bodily sensations and mental states during exercise, while compassion adds the active element of responding to difficulties (e.g., fatigue, self-doubt) with kindness and self-soothing rather than criticism. Therefore, incorporating this dynamic, state-like quality of MCA-PA linked to physical activity in the EXSEM-SC would shift the focus from simply “achieving” in physical activity to embracing the process. This shift could serve as a crucial mechanism for enhancing both direct and indirect pathways from physical activity to self-compassion and well-being.

### Research purpose and hypothesis

In this study, we hypothesized that,

1. MCA-PA and self-compassion would mediate the relationship between physical activity and well-being.
2. Different level of physical activity is associated with the variables at various levels of effect.
3. A direct pathway is expected between MCA-PA and well-being.

The current study, therefore, aims to investigate the relationship between physical activity, the levels of Mindful and Compassionate Awareness in Physical Activity (measured by the MCA-PA), self-compassion, and well-being. By examining the underlying mechanisms and potential pathways involved, this study seeks to contribute to a better understanding of the role of mindfulness and self-compassion in physical activity and well-being, thus providing insights into the development of effective interventions.

## Methodology

### Study design

This cross‑sectional study used path analysis to investigate the associations among physical activity, the Mindful and Compassionate Awareness Scale for Physical Activity, self‑compassion, and well‑being. The reporting of this study followed the *Strengthening the Reporting of Observational Studies in Epidemiology* (STROBE) Statement (Von Elm et al., 2008). Ethical approval was obtained from the Education University of Hong Kong (Ref: 2022‑2023‑0501), and the study was conducted in accordance with the Declaration of Helsinki. Informed consent was obtained online from all participants prior to their inclusion in the study.

### Participants and setting

Hong Kong adolescents aged 12–18 were recruited through convenience sampling at local secondary schools in Hong Kong during the first semester 1 of the 2024–2025 school term. Students with any kind of mental disorder were excluded from the study. Online questionnaires were distributed through the schoolteachers during physical education lessons, regardless of their physical activity level. According to the rule of thumb for conducting structural equation modelling of 1:10, approximately 400 samples are needed for the current study.

### Measurement

#### Self-compassion

The Chinese version of the Self‑Compassion Scale for Youth (SCS‑Youth; Huang et al., 2022; Neff et al., 2021) will be used to assess self‑compassion. This scale comprises 17 items reflecting six components of self‑compassion: self‑kindness, common humanity, mindfulness, self‑judgement, isolation, and over‑identification. Each item is rated on a 5‑point scale ranging from 1 (almost never) to 5 (almost always). The SCS‑Youth has demonstrated high internal consistency (Cronbach’s alpha values typically > .80 for the total scale and subscales), indicating strong reliability. The scale has been validated and shown to possess adequate reliability and validity within the Chinese population.

#### The Godin-Shephard Leisure-Time Physical Activity Questionnaire

The Godin – Shephard Leisure‑Time Physical Activity Questionnaire is a validated four‑item tool for self‑assessment of exercise frequency over the previous week (Godin, 2011). Widely used in health research (Amireault et al., 2015), it evaluates three exercise intensities: strenuous, moderate, and light. The overall leisure‑time activity score is calculated by multiplying the frequency of strenuous exercise by 9, moderate exercise by 5, and light exercise by 3, with higher scores reflecting greater physical activity levels during the past week. The Godin – Shephard questionnaire demonstrates acceptable test – retest reliability and good internal consistency across various populations, including diverse age groups and cultural contexts (Amireault et al., 2015).

#### The World Health Organization- Five Well-Being Index (WHO-5) – Chinese version

The World Health Organization’s Five Well‑Being Index (WHO‑5) – Chinese version (Bech et al., 2003) is used to assess participants’ psychological well‑being, focusing on perceived happiness and positive mood using a 6‑point Likert scale (0 = at no time to 5 = all of the time). The WHO‑5 is recognised as an effective tool for evaluating mental health status, demonstrating a sensitivity of 0.89 and a specificity of 0.87 in adolescent depression screening (Allgaier et al., 2012). Analyses of the Chinese version have also shown strong psychometric properties, including an internal consistency of 0.85 and satisfactory construct validity (Chan et al., 2022; Fung et al., 2022), confirming the reliability and validity of the WHO‑5 for use with Chinese populations.

#### Mindful and Compassionate Awareness Scale for Physical Activity (MCA-PA)

The MCA‑PA was developed to integrate self‑compassion into the existing State Mindfulness Scale for Physical Activity (SMS‑PA‑2) and to refine the scale by incorporating elements of compassion, including self‑compassion and body compassion. The scale aims to highlight the importance of understanding individuals’ experiences of mindfulness and compassion during physical activity. It was tested among more than 500 individuals, including both adults and adolescents, and demonstrated strong psychometric properties, with Cronbach’s alpha coefficients greater than .80 and construct validity exceeding .90. These findings indicate that the MCA‑PA is a valid and reliable instrument for assessing mindfulness and compassion in the context of physical activity, suggesting its potential to support the promotion of self‑compassion in daily physical activities (Wong et al., under review).

### Data analysis

SPSS 28 was used to enter participants’ demographic information, including age and gender. Descriptive statistics were conducted to summarize data on physical activity, self‑compassion, well‑being, and mindful compassionate awareness related to exercise levels. Pearson correlations were computed to examine the basic relationships among these variables. Confirmatory factor analysis (CFA) and path analysis were performed in LISREL 12 using the full‑information maximum likelihood method for model estimation. Model fit was evaluated using several indicators: a comparative fit index (CFI) and a non‑normed fit index (NNFI) greater than 0.90, a standardized root mean square residual (SRMR) of 0.08 or less, and a root mean square error of approximation (RMSEA) of 0.08 or lower, with a 90% confidence interval within these bounds indicating good fit (Hu & Bentler, 1999; Jaccard & Wan, 1996). The Akaike Information Criterion (AIC) was also reported to compare the balance between model fit and complexity. The analyses aimed to identify relationships among the variables and examine potential mediating effects. Additionally, model invariance (configural, metric, and scalar) across gender and perceived mental health status was tested, and a sensitivity analysis was conducted to assess the influence of sample size on the identification of meaningful pathways. The investigation employed the *lavaan* and *simsem* packages in RStudio, using Monte Carlo simulations for structural equation modelling.

## Results

A total of 414 secondary school students (44.9% of females) of a mean age of 14.95 (SD = 1.60) participated in this study. The outcomes of all measurement indicators are normally distributed, with a skewness of approximately 0.12, and no significant outliers are identified. The descriptive statistics and bivariate correlations among the indicators are presented in Table 1.

**Table 1.** Descriptive statistics and correlation matrix of the outcome variables.

	M	SD	MSC-PA	SCY	WHO	PAV	PAM	PAL	PA_Total
MCAPA	2.84	0.87	1.00						
SCY	3.19	0.31	.55**	1.00					
WHO- 5	12.03	6.24	.34**	.38**	1.00				
PAV	19.95	17.05	.19**	0.06	.16**	1.00			
PAM	9.84	9.57	.13**	0.01	0.08	.67**	1.00		
PAL	5.03	5.88	.11*	0.06	0.07	.43**	.61**	1	
PA_Total	34.80	28.11	0.16**	0.05	0.14**	0.92**	0.87**	0.68**	1

Note. **Correlation is significant at the 0.01 level (2-tailed).

MCA-PA – Mindful and Compassion Awareness for Physical Activity; SCY – Self-compassion – Youth; WHO – World Health Organization Five Well-being Index; PAV – Physical Activity – Vigorous; PAM – Physical Activity – Moderate; PAL – Physical Activity – Light; PA_Total – Total Physical Activity Level.

### Reliability and validity of the measurement indicators

The measurement models demonstrated strong psychometric properties (Table 2). The MCA‑PA showed excellent factorial validity (Kaiser – Meyer – Olkin [KMO] = 0.97) and internal consistency (α = .98), supported by acceptable fit indices (CFI = 0.98, RMSEA = 0.13, SRMR = 0.03). Similarly, the SCY demonstrated robust factorial validity (KMO = 0.96) and adequate reliability (α = .81), along with an acceptable model fit (CFI = 0.94, RMSEA = 0.16, SRMR = 0.07). The WHO‑5 also demonstrated high factorial validity (KMO = 0.91) and internal consistency (α = .96), with acceptable fit indices (CFI = 0.98, RMSEA = 0.16, SRMR = 0.02), supporting its suitability for subsequent path analysis. Although the physical activity measure did not provide factorial validity data, it demonstrated acceptable reliability (α = .80). Given the established validity and reliability of these measurement instruments, path analysis was conducted to examine the hypothesized structural relationships among the latent constructs.

**Table 2.** Goodness-of-fit indices for measurement instruments.

Fit Index	MCA-PA	SCY	WHO-5
RMSEA	0.12	0.16	0.16
90% CI for RMSEA	(0.12–0.13)	(0.15–0.17)	(0.12–0.19)
NNFI (TLI)	0.97	0.92	0.97
CFI	0.98	0.91	0.98
Standardized RMR	0.027	0.067	0.016
Model AIC	1368.15	1065.84	75.06

Note. RMSEA – Root Mean Square Error of Approximation; NNFI (TLI) – Non-Normed Fit Index (also known as Tucker-Lewis Index); CFI – Comparative Fit Index; Standardized RMR – Standardized Root Mean Square Residual; Model AIC – Model Akaike Information Criterion; MCA-PA – Mindful and Compassion Awareness for Physical Activity; SCY – Self-compassion – Youth; WHO – World Health Organization Five Well-being Index.

### Path analysis

**Figure 1.**
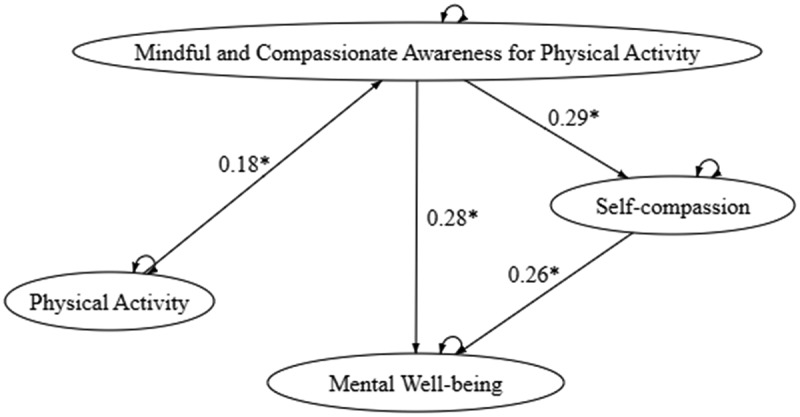
Model 1 - Path analysis model of overall physical activity and self-compassion.

**Figure 2.**
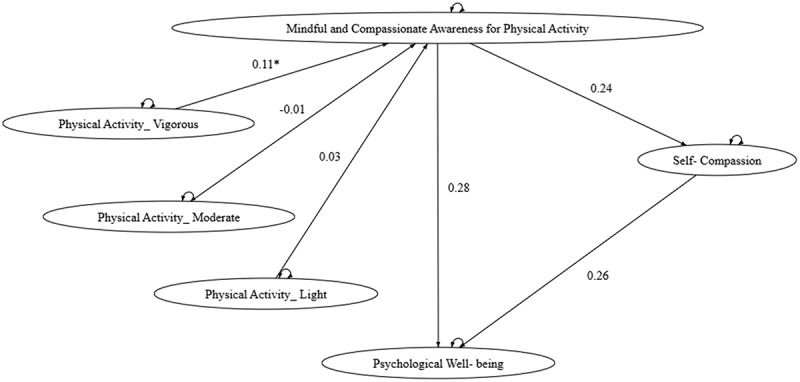
Model 2 - Path analysis of different level of physical activity and self-compassion.

**Figure 3.**
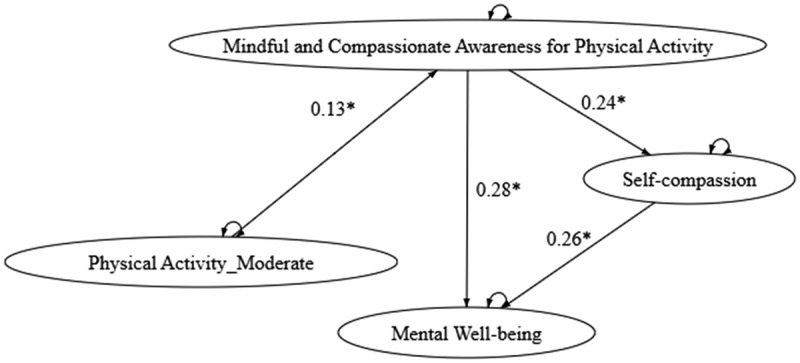
Model 2.1 - Path analysis model of moderate-intensity physical activity and self-compassion.

**Figure 4.**
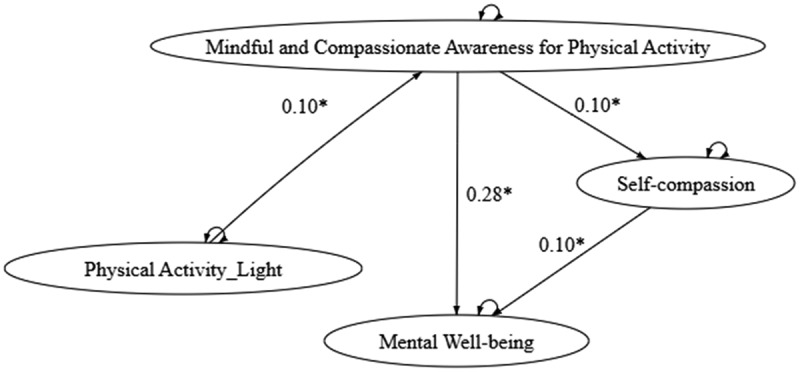
Model 2.2 - Path analysis model of light-intensity physical activity and self-compassion.

Model 1 examines the direct and mediated relationships between physical activity and psychological well‑being through MCA‑PA and self‑compassion. The fit indices indicate that the model adequately represents the proposed relationships (see Figure 1 and Column 1 of Table 3). Model 2 (Figure 2; Column 2 of Table 3) explores how different levels of physical activity intensity – high (PAV), moderate (PAM), and low (PAL) – influence psychological well‑being, with MCA‑PA and self‑compassion as mediators. Notably, the path from high‑intensity physical activity to MCA‑PA emerged as the only significant pathway leading to self‑compassion and psychological well‑being. When examining light and moderate intensities separately, Model 2.1 and Model 2.2 (Figures 3 and 4; Columns 3 and 4 of Table 3) each showed a significant path from PAM or PAL to MCA‑PA.

**Table 3.** Goodness-of-fit indices for the outcome models.

Fit Index	Model 1	Model 2	Model 2.1	Model 2.2
RMSEA	0.065	0.028	0.022	0.028
90% CI for RMSEA	(0.0; 0.13)	(0.0; 0.074)	(0.0; 0.10)	(0.0; 0.11)
NNFI (TLI)	0.92	0.99	0.99	0.98
CFI	0.97	1.00	1.00	0.99
Standardized RMR	0.039	0.038	0.026	0.026
Model AIC	21.52	37.93	18.38	18.65

Note. RMSEA – Root Mean Square Error of Approximation; NNFI (TLI) – Non-Normed Fit Index (also known as Tucker-Lewis Index); CFI – Comparative Fit Index; Standardized RMR – Standardized Root Mean Square Residual; Model AIC – Model Akaike Information Criterion; MCA-PA – Mindful and Compassion Awareness for Physical Activity; SCY – Self-compassion – Youth; WHO – World Health Organization Five Well-being Index.

Across all models, the consistent and significant direct path from MCA‑PA to well‑being underscores the transformative role of mindful engagement in physical activity. The robustness of the model across varying activity levels and intensities confirms the positive impact of physical activity on mental health, achieved through the cultivation of mindful and compassionate awareness during exercise, which in turn promotes self‑compassion. The goodness‑of‑fit indices for all models are presented in Table 3.

Structural invariance was tested across gender groups and across levels of perceived happiness and positive mood (ranging from 1 = poor to 5 = excellent). Chi‑square difference tests were used to evaluate configural, metric, and scalar invariance, providing insight into the stability of factor loadings and intercepts across groups.

### Gender invariance

For Model 1, the chi‑square difference tests comparing gender groups (male vs. female) showed no significant differences in metric invariance (χ^2^ diff = 0.00, RMSEA = 0.00) and a non‑significant scalar invariance test (χ^2^ diff = 5.60, RMSEA = 0.07, *p* = .13). These results indicate that the model is invariant across gender, suggesting that it fits similarly for both male and female participants.

Model 2, including its variations (Model 2.1 and Model 2.2), also demonstrated gender invariance. All models showed no significant differences in metric invariance (χ^2^ diff = 0.00, RMSEA = 0.00), and although Model 2 exhibited a higher scalar invariance value, it remained non‑significant (χ^2^ diff = 27.20, RMSEA = 0.00, *p* = .61). Model 2.1 and Model 2.2 also showed non‑significant scalar invariance results (χ^2^ diff = 4.50, RMSEA = 0.05, *p* = .21 for both models).

Overall, the invariance tests across all models consistently indicate that the factor structure is stable across gender. The non‑significant chi‑square differences in scalar invariance further suggest that the intercepts of the observed variables do not differ across groups, reinforcing the applicability of the models across diverse populations.

## Discussion

The primary objective of this study was to explore the intricate relationships between physical activity, mindfulness and compassion as measured by the MCA-PA, self-compassion, and overall well-being. Our findings substantiate the hypothesized mediating roles of MCA-PA and self-compassion, elucidating how these factors interact to influence well-being in the context of physical activity. Specifically, the results confirm that MCA-PA and self-compassion effectively mediate the relationship between physical activity and well-being, especially among Hong Kong secondary school students.

Responding to hypothesis 1, the first model showed a good fit model, supporting the theory that physical activity not only directly affects psychological well-being but also does so indirectly by encouraging conscious self-care practices and improving self-compassion. The important route from MCA-PA to well-being emphasizes the need to include mindfulness in physical exercise for psychological advantages. This outcome has further expanded the EXSEM-SC, as well as again evidenced among Hong Kong secondary school students. A study of college students found a significant relationship between mindfulness and enhanced eudaimonic well-being, which in turn contributed to increased physical activity (S. Zhang et al., 2023).

Referring to Hypothesis 2, the second model suggests that high‑intensity physical activity (e.g., running, jogging, vigorous swimming) supports mindfulness and self‑care practices as effectively as moderate‑intensity (e.g., fast walking, easy bicycling) or low‑intensity (e.g., yoga, easy walking) exercise. This indicates that high‑intensity activity alone is positively related to mindfulness and self‑care when examined independently. However, the non‑significant pathways for PAM and PAL reveal a suppression effect among the physical activity intensity variables. When all intensities are considered simultaneously, vigorous physical activity emerges as the significant predictor, likely because it serves as a strong indicator of overall physical activity, engagement and lifestyle. Nevertheless, when focusing specifically on low and moderate physical activity, Models 2.1 and 2.2 show significant pathways from PAM and PAL to MCA‑PA. This suggests that modest levels of exercise can help individuals strike a balance, promoting greater mindfulness and self‑care. Low‑intensity activities may also facilitate enhanced mindfulness due to their less demanding nature. For example, combining moderate‑intensity walking with mindful awareness has been shown to increase state mindfulness in both bodily and mental domains compared with physical activity alone (Solk et al., 2023). Similarly, participants in a mindfulness‑based treatment group demonstrated more positive affective responses to exercise than those in associative concentration condition (Gillman & Bryan, 2020). These findings support the proposition that mindful awareness during physical activity reduces perceived effort, making the exercise experience more pleasant.

Physical activity also creates immediate opportunities for cultivating self‑compassion through mindfulness and present‑moment awareness. During exercise, individuals often engage in contemplation and self‑reflection, particularly during solo activities such as running, walking, or basketball. This process facilitates the development of mindfulness, one of the three core components of self‑compassion, alongside self‑kindness and common humanity (Wong et al., 2023), and aligns with the pathways demonstrated empirically in the EXSEM‑SC model.

### Limitations and future implications

The current study represents a novel investigation into the direct and indirect relationships among physical activity, mindful and compassionate awareness of physical activity, and self‑compassion in Hong Kong secondary school students. It highlights the importance of cultivating inner awareness during physical activity, focusing not only on bodily sensations but also on broader internal experiences. This study also addresses a gap in the literature, as most existing research has examined the effects of self‑compassion on physical activity motivation, self‑regulation, athletic performance, and other exercise‑related outcomes. Nonetheless, emotional regulation shows strong potential as a moderator within the EXSEM‑SC pathway. Research indicates that emotional regulation functions as a key mechanism linking physical activity to psychological outcomes (Wang et al., 2025). Individuals with stronger emotional regulation skills may derive greater mindfulness and self‑care benefits from physical activity because they are better able to process and integrate affective experiences during exercise (Fuentealba-Urra et al., 2023). Moreover, high‑intensity activity emerges as a significant predictor when all intensities are considered simultaneously. Evidence suggests that vigorous activities present greater opportunities for perceived failure or difficulty, and individuals higher in self‑compassion demonstrate better emotional recovery from such challenges. Thus, students’ typical responses to athletic challenges or perceived failures during high‑intensity activity may moderate whether such activity enhances or undermines mindful self‑care practices (S. Zhang et al., 2023). Overall, these findings underscore the need for further longitudinal and experimental research to establish causal pathways and to expand the EXSEM‑SC model by incorporating potential moderators such as emotional regulation and perceived athletic experiences.

This study is cross‑sectional; it does not establish the causal effects, or intervention impacts of physical activity, nor does it examine the relationships between different levels of physical activity and MCA‑PA or self‑compassion. Notably, a 12‑week low‑intensity exercise program combined with psychoeducation produced significant outcomes in women with metabolic syndrome, reducing stress levels by 23% and depressive symptoms by 37%. This integrated approach highlights the combined effects of moderate physical activity and mental health education on psychological well‑being (Morga et al., 2021). In a pilot study conducted by the author (currently under review), an 8‑week Mindful Self‑Compassion Exercise (MSCE) program led Hong Kong secondary school students to engage in moderate‑intensity physical activities, such as aerobic fitness and dancing, which had a stronger impact on their MCA‑PA. Conversely, participation in low‑intensity physical activities, such as yoga, was associated with greater mental relaxation and self‑compassion. Therefore, a randomized controlled trial should be conducted to replicate the 8‑week MSCE pilot study, comparing the effects of exercise alone versus exercise combined with psychoeducation on MCA‑PA, self‑compassion, and psychological well‑being.

Although the findings offer valuable insights, the generalizability of the results is limited by the sample, which consisted solely of adolescents from Hong Kong. Despite evidence of structural invariance across gender and mental health status, cultural and contextual factors may restrict broader applicability. Future research involving more diverse populations is needed to enhance external validity and support the wider application of the MCA-PA model. This comprehensive modelling approach facilitates a deeper understanding of the mechanisms at play. It promotes a mindful and compassionate awareness of physical activity as a valuable tool for enhancing psychological well-being. Additionally, increasing evidence highlights the prevalence of mental health issues among secondary school students, along with the significant influence of physical activity on mental health. Future research aimed at understanding how physical activity affects MCA-PA and self-compassion will be essential. Health promotion programs in college settings and other contexts should consider incorporating mindfulness and self-compassion into their offerings as a means of enhancing well-being and promoting healthy behaviours. Promoting psychoeducation, particularly mindful self-compassion, alongside physical activity prescriptions, is essential for alleviating mental illness and supporting psychological well-being.

## Supplementary Material

items tables in appendix.docx

## Data Availability

All data generated or analysed during this study can be provided upon request.
