# Supplementary material for: Investigating the relationships between physical activity, mindful self-compassion, and well-being among Chinese adolescents: the Exercise Self-Esteem Model Revised with Self-Compassion 2.0
Source: Aust J Psychol. 2026 Feb 1;78(1):2620254. doi: 10.1080/00049530.2026.2620254 (PMC12862845; doi:10.1080/00049530.2026.2620254)
Supplement: items tables in appendix.docx [file RAUP_A_2620254_SM1662.docx]

**MCA-PA**

|  | 1  Not at all | 2  A little | 3  Moderately | 4  Quite a bit | 5  Very much |
| --- | --- | --- | --- | --- | --- |
| I was aware of the different feelings that came into me during physical activity. |  |  |  |  |  |
| I noticed pleasant and unpleasant emotion/thoughts during physical activity. |  |  |  |  |  |
| I allow myself to be fierce during physical activity, when I notice unpleasant feelings/thoughts (i.e., anger, allowing yourself to vent or express your negative feelings). |  |  |  |  |  |
| I give myself with care and love through physical activity, when noticing unpleasant emotions/thoughts. |  |  |  |  |  |
| When I notice unpleasant thoughts/emotions, physical activity makes it possible for me not to blow these painful experiences out of proportion. |  |  |  |  |  |
| It was interesting to see the patterns of my thinking during physical activity. |  |  |  |  |  |
| I focused on the movement of my body during physical activity. |  |  |  |  |  |
| I felt present in my body during physical activity. |  |  |  |  |  |
| I listened to what my body was telling me during physical activity. |  |  |  |  |  |
| I was aware of how my body feel during physical activity. |  |  |  |  |  |
| I noticed the sensations in my body during physical activity. |  |  |  |  |  |
| I was aware of my muscle strength/aerobic ability during physical activity. |  |  |  |  |  |
| During physical activity, it allows me not to be obsessed with or/ and fixate on unpleasant thoughts/emotions. |  |  |  |  |  |
| During physical activity, it enables me to accept my thoughts/emotions without judging them. |  |  |  |  |  |
| When I notice unpleasant thoughts/emotions, doing physical activity allows me to recognize that feelings of inadequacy are common to most people. |  |  |  |  |  |
| When I notice unpleasant thoughts/emotions, physical activity makes it possible for me not to feel alone in failure or deficiency. |  |  |  |  |  |
| During physical activity, I acknowledged how my body felt without trying to change it. |  |  |  |  |  |
| I accepted how my body felt even if it was unpleasant. |  |  |  |  |  |
| I tend not to isolate how my body felt, when it did not meet expectations during physical activity. |  |  |  |  |  |
| I try to take a balanced view between my expectations and my body's inability during physical activity. |  |  |  |  |  |

**Self-Compassion Scale for Youth**

|  | 1 Almost never | 2  Not very often | 3 Sometimes | 4  Very often | 5 Almost always |
| --- | --- | --- | --- | --- | --- |
| 1. I try to be kind and supportive to myself when I’m having a hard time. |  |  |  |  |  |
| 1. When I feel sad or down, it seems like I'm the only one who feels that way. |  |  |  |  |  |
| 1. When I notice things about myself that I don’t like, I get really frustrated. |  |  |  |  |  |
| 1. When I feel I’m not “good enough” in some way, I try to remind myself that other people sometimes feel this way too. |  |  |  |  |  |
| 1. When I feel frustrated or disappointed, I think about it over and over again. |  |  |  |  |  |
| 1. When something upsetting happens I try to see things as they are without blowing it out of proportion. |  |  |  |  |  |
| 1. I get mad at myself for not being better at some things. |  |  |  |  |  |
| 1. When I’m sad or unhappy, I remember that other people also feel this way at times. |  |  |  |  |  |
| 1. I’m kind to myself when things go wrong and I’m feeling bad. |  |  |  |  |  |
| 1. When I feel bad or upset, I tend to feel most other people are probably happier than I am |  |  |  |  |  |
| 1. When something difficult happens, I try to see things clearly without exaggerations. |  |  |  |  |  |
| 1. I’m really hard on myself when I do something wrong. |  |  |  |  |  |
| 1. When things aren’t going well, I keep in mind that life is sometimes hard for everyone. |  |  |  |  |  |
| 1. When I’m feeling bad or upset, I can’t think of anything else at the time. |  |  |  |  |  |
| 1. I try to be understanding and patient with myself even when I mess up. |  |  |  |  |  |
| 1. When I’m really struggling, I tend to feel like other people are probably having an easier time of it. |  |  |  |  |  |
| 1. When something upsets me, I try to notice my feelings and not get carried away by them. |  |  |  |  |  |

**Godin-Shephard Leisure-Time Physical Activity Questionnaire**

During a typical **7-Day period** (a week), how many times on average do you do the following kinds of exercise for **more than 15 minutes** during your free time (write on each line the appropriate number).

**Weekly leisure activity score** = (9 × Strenuous) + (5 × Moderate) + (3 × Light)

|  | Times per week |  | Totals |
| --- | --- | --- | --- |
| a) **STRENUOUS EXERCISE (HEART BEATS RAPIDLY)** (e.g., running, jogging, hockey, football, soccer, squash, basketball, cross country skiing, judo, roller skating, vigorous swimming, vigorous long distance bicycling) |  | X9 |  |
| b) **MODERATE EXERCISE (NOT EXHAUSTING)** (e.g., fast walking, baseball, tennis, easy bicycling, volleyball, badminton, easy swimming, alpine skiing, popular and folk dancing |  | X5 |  |
| C) **MILD/LIGHT EXERCISE (MINIMAL EFFORT)** (e.g., yoga, archery, fishing from river bank, bowling, horseshoes, golf, snow-mobiling, easy walking |  | X3 |  |
| **WEEKLY LEISURE-TIME ACTIVITY SCORE** |  |  |  |

EXAMPLE

Strenuous = 3 times/wk

Moderate = 6 times/wk

Light = 14 times/wk

Total leisure activity score = (9 × 3) + (5 × 6) + (3 × 14) = 27 + 30 + 42 = 99

| **Godin Scale Score** | **Interpretation** |
| --- | --- |
| 24 units or more | Active |
| 14 – 23 units | Moderately Active |
| Less than 14 units | Insufficiently Active/Sedentary |

**WHO-5 Well-being Index**

| Please respond to each item by marking one box per row, regarding how you felt in the last two weeks. | | All of the time | Most of the time | More than half the time | Less than half the time | Some of the time | At no time |
| --- | --- | --- | --- | --- | --- | --- | --- |
| WHO 1 | I have felt cheerful in good spirits. |  |  |  |  |  |  |
| WHO 2 | I have felt calm and relaxed. |  |  |  |  |  |  |
| WHO 3 | I have felt active and vigorous. |  |  |  |  |  |  |
| WHO 4 | I woke up feeling fresh and rested. |  |  |  |  |  |  |
| WHO 5 | My daily life has been filled with things that interest me. |  |  |  |  |  |  |

Scoring: The raw score is calculated by totaling the figures of the five answers. The raw score ranges from 0 to 25, 0 representing worst possible and 25 representing best possible quality of life.

To obtain a percentage score ranging from 0 to 100, the raw score is multiplied by 4. A percentage score of 0 represents worst possible, whereas a score of 100 represents best possible quality of life.

**Previous Questionnaires including in the EXSEM-SC model**

**PAQ-A Scale Physical Activity Questionnaire for Adolescents (PAQ‑A)**

**Scale:**
1 = Low physical activity  2 = Slightly active  3 = Moderately active  4 = Active  5 = Very active

| **Item No.** | **Statement** | **1** | **2** | **3** | **4** | **5** |
| --- | --- | --- | --- | --- | --- | --- |
| 1 | In your spare time over the last 7 days, how often did you do sports, dance, or play games that required physical activity? | ☐ | ☐ | ☐ | ☐ | ☐ |
| 2 | During physical education (PE) classes in the last 7 days, how often were you very active (playing hard, running, jumping, throwing)? | ☐ | ☐ | ☐ | ☐ | ☐ |
| 3 | In the last 7 days, what did you normally do at lunch (besides eating lunch)? | ☐ | ☐ | ☐ | ☐ | ☐ |
| 4 | In the last 7 days, how often did you do sports, dance, or play games after school? | ☐ | ☐ | ☐ | ☐ | ☐ |
| 5 | In the last 7 days, how often did you do sports, dance, or play games in the evenings? | ☐ | ☐ | ☐ | ☐ | ☐ |
| 6 | On the last weekend, how many times did you do sports, dance, or play games? | ☐ | ☐ | ☐ | ☐ | ☐ |
| 7 | Which best describes your activity during your free time last week? (e.g., watching TV, walking, biking, playing sports) | ☐ | ☐ | ☐ | ☐ | ☐ |
| 8 | In the last 7 days, how often did you do physical activity so hard that you got out of breath, or sweated? | ☐ | ☐ | ☐ | ☐ | ☐ |
| 9 | Describe the frequency of your physical activity each day last week (Mon–Sun). | ☐ | ☐ | ☐ | ☐ | ☐ |

**Exercise Self-efficacy Scale**

**Scale:**
0 = Not at all confident  50 = Moderately confident  100 = Highly confident
*(Responses are given in 10‑point increments: 0, 10, 20 … 100)*

| **Item No.** | **Statement** | **0** | **10** | **20** | **30** | **40** | **50** | **60** | **70** | **80** | **90** | **100** |
| --- | --- | --- | --- | --- | --- | --- | --- | --- | --- | --- | --- | --- |
| 1 | I am able to exercise 3 times per week at moderate intensity for 40+ minutes without quitting for the next month. | ☐ | ☐ | ☐ | ☐ | ☐ | ☐ | ☐ | ☐ | ☐ | ☐ | ☐ |
| 2 | I am able to exercise 3 times per week at moderate intensity for 40+ minutes without quitting for the next 2 months. | ☐ | ☐ | ☐ | ☐ | ☐ | ☐ | ☐ | ☐ | ☐ | ☐ | ☐ |
| 3 | I am able to exercise 3 times per week at moderate intensity for 40+ minutes without quitting for the next 3 months. | ☐ | ☐ | ☐ | ☐ | ☐ | ☐ | ☐ | ☐ | ☐ | ☐ | ☐ |
| 4 | I am able to exercise 3 times per week at moderate intensity for 40+ minutes without quitting for the next 4 months. | ☐ | ☐ | ☐ | ☐ | ☐ | ☐ | ☐ | ☐ | ☐ | ☐ | ☐ |
| 5 | I am able to exercise 3 times per week at moderate intensity for 40+ minutes without quitting for the next 5 months. | ☐ | ☐ | ☐ | ☐ | ☐ | ☐ | ☐ | ☐ | ☐ | ☐ | ☐ |
| 6 | I am able to exercise 3 times per week at moderate intensity for 40+ minutes without quitting for the next 6 months. | ☐ | ☐ | ☐ | ☐ | ☐ | ☐ | ☐ | ☐ | ☐ | ☐ | ☐ |
| 7 | I am able to exercise 3 times per week at moderate intensity for 40+ minutes without quitting for the next 7 months. | ☐ | ☐ | ☐ | ☐ | ☐ | ☐ | ☐ | ☐ | ☐ | ☐ | ☐ |
| 8 | I am able to exercise 3 times per week at moderate intensity for 40+ minutes without quitting for the next 8 months. | ☐ | ☐ | ☐ | ☐ | ☐ | ☐ | ☐ | ☐ | ☐ | ☐ | ☐ |
| 9 | I am able to exercise 3 times per week at moderate intensity for 40+ minutes without quitting for the next 9 months. | ☐ | ☐ | ☐ | ☐ | ☐ | ☐ | ☐ | ☐ | ☐ | ☐ | ☐ |
| 10 | I am able to exercise 3 times per week at moderate intensity for 40+ minutes without quitting for the next 10 months. | ☐ | ☐ | ☐ | ☐ | ☐ | ☐ | ☐ | ☐ | ☐ | ☐ | ☐ |

**Body Compassion Scale**

**Scale:**
1 = Almost Never  2 = Rarely  3 = Sometimes  4 = Often  5 = Almost Always

| **Item** | **Subscale** | **Statement** | **1** | **2** | **3** | **4** | **5** |
| --- | --- | --- | --- | --- | --- | --- | --- |
| 1 | Defusion | I can step back from judging my body harshly. |  |  |  |  |  |
| 2 | Defusion | I notice body-related thoughts without getting caught up in them. |  |  |  |  |  |
| 3 | Defusion | I can separate myself from negative thoughts about my body. |  |  |  |  |  |
| 4 | Defusion | I can observe my body thoughts without reacting. | ☐ | ☐ | ☐ | ☐ | ☐ |
| 5 | Defusion | I can distance myself from critical thoughts about my body. | ☐ | ☐ | ☐ | ☐ | ☐ |
| 6 | Defusion | I can see body thoughts as just thoughts. | ☐ | ☐ | ☐ | ☐ | ☐ |
| 7 | Defusion | I can let go of unhelpful body judgments. | ☐ | ☐ | ☐ | ☐ | ☐ |
| 8 | Defusion | I can recognize body thoughts without believing them. | ☐ | ☐ | ☐ | ☐ | ☐ |
| 9 | Common Humanity | I recognize that many people struggle with body image. | ☐ | ☐ | ☐ | ☐ | ☐ |
| 10 | Common Humanity | I remind myself I am not alone in experiencing body dissatisfaction. | ☐ | ☐ | ☐ | ☐ | ☐ |
| 11 | Common Humanity | I see body challenges as part of being human. | ☐ | ☐ | ☐ | ☐ | ☐ |
| 12 | Common Humanity | I realize others also feel negatively about their bodies sometimes. | ☐ | ☐ | ☐ | ☐ | ☐ |
| 13 | Common Humanity | I understand body struggles are common. | ☐ | ☐ | ☐ | ☐ | ☐ |
| 14 | Common Humanity | I feel connected to others who have body image concerns. | ☐ | ☐ | ☐ | ☐ | ☐ |
| 15 | Common Humanity | I acknowledge body image difficulties are shared by many. | ☐ | ☐ | ☐ | ☐ | ☐ |
| 16 | Acceptance | I accept my body as it is right now. | ☐ | ☐ | ☐ | ☐ | ☐ |
| 17 | Acceptance | I treat my body with kindness even when I wish it looked different. | ☐ | ☐ | ☐ | ☐ | ☐ |
| 18 | Acceptance | I can live with imperfections in my body. | ☐ | ☐ | ☐ | ☐ | ☐ |
| 19 | Acceptance | I respect my body despite flaws. | ☐ | ☐ | ☐ | ☐ | ☐ |
| 20 | Acceptance | I am gentle with my body when I feel dissatisfied. | ☐ | ☐ | ☐ | ☐ | ☐ |
| 21 | Acceptance | I can appreciate my body even if it doesn’t meet ideals. | ☐ | ☐ | ☐ | ☐ | ☐ |
| 22 | Acceptance | I value my body regardless of appearance. | ☐ | ☐ | ☐ | ☐ | ☐ |
| 23 | Acceptance | I accept limitations of my body. | ☐ | ☐ | ☐ | ☐ | ☐ |
